# Homocysteine induces mitochondrial dysfunction involving the crosstalk between oxidative stress and mitochondrial pSTAT3 in rat ischemic brain

**DOI:** 10.1038/s41598-017-07112-z

**Published:** 2017-07-31

**Authors:** Shuang Chen, Zhiping Dong, Yaqian Zhao, Na Sai, Xuan Wang, Huan Liu, Guowei Huang, Xumei Zhang

**Affiliations:** Department of Nutrition and Food Science, School of Public Health, Tianjin Medical University, Tianjin, China

## Abstract

Homocysteine (Hcy) has been shown to have a neurotoxic effect on ischemic brain cells; however, the underlying mechanisms remain incompletely understood. Here, we examined whether Hcy treatment influences mitochondria injury, oxidative stress, and mitochondrial STAT3 (mitoStat3) expression in rat ischemic brain. Our results demonstrated that Hcy treatment aggravated the damage of mitochondrial ultrastructure in the brain cortex and the dentate gyrus region of the hippocampus after focal cerebral ischemia. An elevated Hcy level was also accompanied by the significant inhibition of mitochondrial complex I–III enzymatic activities in addition to an increase in cytochrome c release. 8-Hydroxy-2′-deoxyguanosine (8-OHdG) content and mitoStat3 protein phosphorylation level were increased in Hcy-treated animals, whereas AG490, a Jak2 inhibitor, inhibited mitoStat3 phosphorylation as well as 8-OHdG levels induced by Hcy. *In vitro* studies revealed that Hcy also markedly increased reactive oxygen species (ROS) and mitoStat3 levels. In addition, the inhibition of pSTAT3 reduced Hcy-mediated increase in ROS levels, whereas quenching ROS using the ROS inhibitor glutathione ethyl ester inhibited Hcy-mediated pSTAT3 overactivation in Neuro2a cells. These findings suggest that the development of therapies that interfere with the ROS/pSTAT3 pathway may be helpful for treating cerebral infarction-related diseases associated with Hcy.

## Introduction

Cerebral ischemic stroke represents a life threatening neurological disorder that leads to mortality and long-term disability in surviving patients. Notably, elevated plasma homocysteine (Hcy) levels have been indicated as a strong and modifiable risk factor of ischemic stroke^1^. In recent years, although the neurotoxic effects of Hcy have been well documented in several neurodegenerative diseases, including Alzheimer’s disease, Parkinson’s disease, and amyotrophic lateral sclerosis^2–5^, only limited data are available that describe the influence of Hcy on brain cell injury in animal models of ischemic stroke. Our previous study demonstrated that homocystinuric rats are highly susceptible to cerebral ischemia. Hcy aggravates neural cell injury in brain cortex region following rat cerebral ischemia-reperfusion (MCAO), and autophagy overactivation which occurs in cortical neurons may be an important mechanism for Hcy neurotoxicity^6^. However, the following several questions still need to be further clarified. Firstly, it is unclear whether neural cell injury caused by Hcy also occurs in the hippocampus, one of the most vulnerable brain regions to ischemic damages. Secondly, the molecular mechanisms of Hcy-induced brain injury are probably complex and multifaceted. In addition to autophagy, what are the other potential mechanisms? Lastly, it was widely accepted that autophagy and mitochondria are elaborately linked homeostatic elements that act in response to changes in cellular environment such as energy, nutrient, and stress^7^. Mitochondrial damage is known to result in activation of autophagy during cerebral ischemia^8^. To our knowledge, it has not previously been reported that mitochondrial damage involved the neurotoxicity of Hcy using a MCAO animal model. The present study on the relationships between Hcy and mitochondrial damage will provide new insight into how Hcy exerts a toxic effect in the brain cortex and hippocampus of the rats after focal ischemia.

Mitochondrial dysfunction plays a vital role in many human diseases because of the important roles these organelles play in cellular metabolism. Several reports have indicated that mitochondria suffer severe damage following cerebral ischemia^9–11^. In addition, evidence has suggested that the generation of reactive oxygen species (ROS) may represent one of the key factors in the induction of mitochondrial damage and dysfunction^12, 13^. After brain ischemia, superoxide anions and ROS are produced in mitochondria. These ROS then induce mitochondrial-dependent apoptotic pathways^14^. Thus, both Hcy and mitochondrial damage are closely related to the pathological process of ischemic stroke. Additionally, Folbergrova *et al*. reported that Hcy results in an accumulation of ROS at high concentrations and inhibits the mitochondrial respiration in rat epilepsy model^15^. However, whether both mitochondrial dysfunction and ROS generation occurred in Hcy-treated ischemic brains has not yet been determined.

In turn, the signal transducers and activators of transcription family 3 (STAT3) protein, which has been historically studied as a signal transduction and transcriptive activation factor, has also been shown by several groups to be easily activated by cerebral ischemic injury, implicating that it may play a vital role in the pathophysiological process of cerebral ischemia and reperfusion injury as well^16–18^. However, in recent years, a small pool of STAT3 has been found to be localized in the mitochondria of non-neuronal cells and to regulate cellular functions independent of its transcriptional activities^19, 20^. Further study showed that the functions of mitochondrial STAT3 (mitoStat3) included cellular metabolism, cell death, development, cancer transformation, ischemia/reperfusion injury of the heart, sperm motility, and T cell immunity^21–25^. However, factors remaining to be determined include the potential for STAT3 activation in mitochondria after cerebral ischemia injury, whether STAT3 is affected by Hcy, and the mechanisms associated with such potential effects in the ischemic brain.

The present study was designed to elucidate the molecular mechanism of neurotoxicity by Hcy in relation to mitochondrial damage, ROS generation, and mitochondrial STAT3 activation in rat ischemic brains. The potential for crosstalk between ROS production and STAT3 activation in the relevant mechanisms during cerebral ischemia-reperfusion injury was also investigated.

## Results

### Mitochondrial ultrastructure

**Figure 1:**
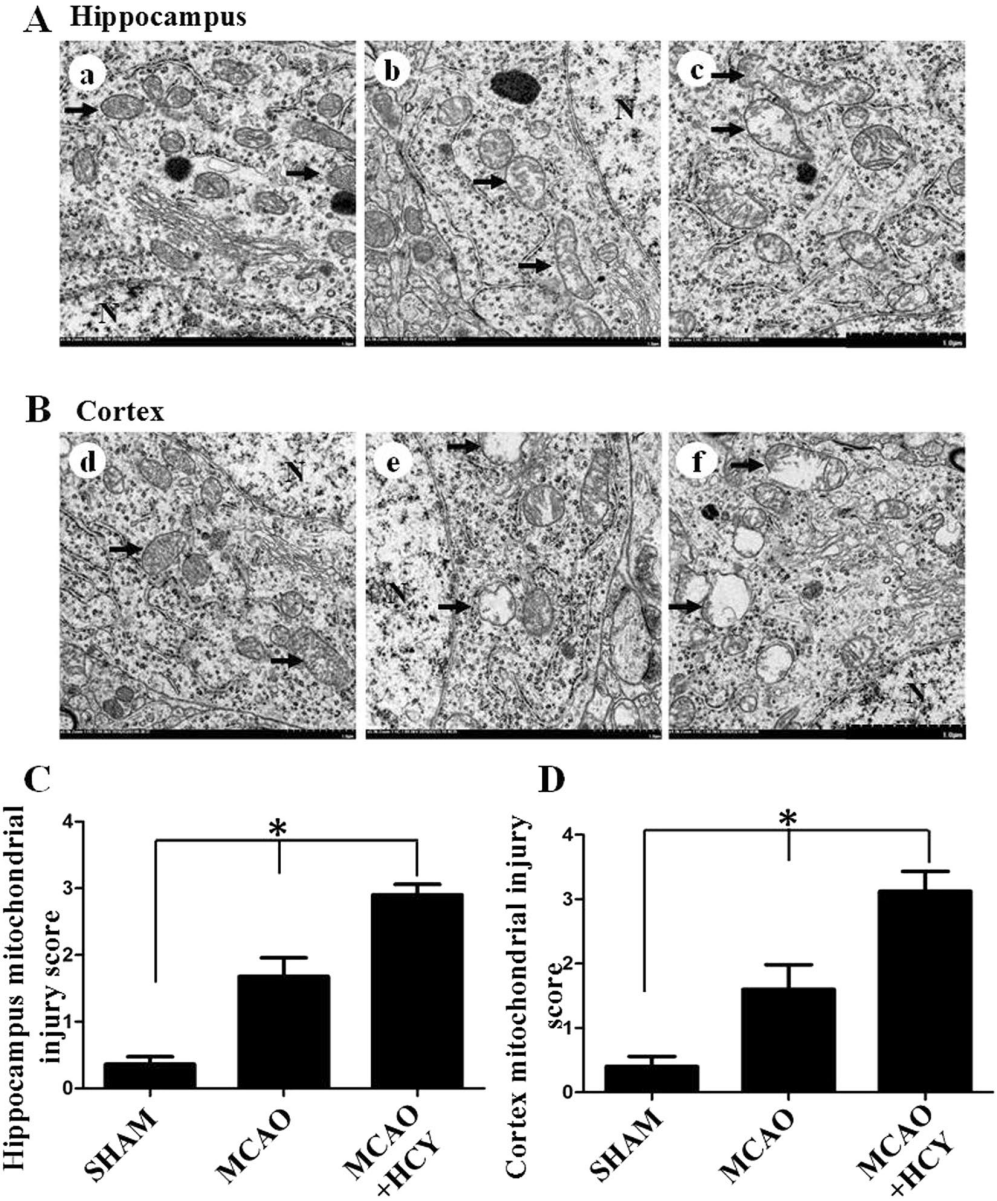
Hcy treatment increased the mitochondrial injury in ischemic brains. Representative electron photomicrographs of mitochondria from the hippocampus (**A**) and brain cortex (**B**). Compared with the normal ultrastructure of mitochondria in the SHAM control animal (a,d), a marked mitochondrial ultrastructure injury was observed at 24 h after MCAO (b and e). After Hcy treatment, the mitochondria demonstrated a range of more serious ultrastructural injuries (arrows). Moderate to severe mitochondrial swelling (c,f) was typical for most of the Hcy-treated animals and high-amplitude swelling with concomitant loss of membrane integrity was also occasionally observed. (**C**,**D**) Injury scores (as determined using the criteria described in the Methods) of brain mitochondria for the SHAM control, MCAO, and MCAO + HCY groups at 24 h post-treatment (values represent the means ± SD). *P < 0.05. Scale bars = 1.0 μm. The arrows indicate the typical changes of mitochondrial morphology.

To investigate the neurotoxic effect of Hcy, D,L-Hcy (1.6 mg/kg/day) was administered by injection 21 d prior to middle cerebral artery occlusion reperfusion operation (MCAO) and up to 24 h post-MCAO (MCAO group). We then examined the mitochondrial morphology change caused by Hcy in the ischemic cerebral tissue by transmission electron microscopy. As shown in Fig. 1A and B, in the sham operation control group (SHAM) group, neurons in the brain cortex and the dentate gyrus (DG) region of the hippocampus exhibited normal nuclei and cytoplasmic contents without signs of edema, nuclear membrane breaks, or clumped tigroid chromatin. Mitochondria also appeared to have normal morphology, demonstrating no evidence of swelling, outer membrane breaks, or intracristal dilation (Fig. 1A,D). In contrast, a marked mitochondrial ultrastructural injury was apparent 24 h after MCAO, compared with that in the SHAM group (Fig. 1B,E). The most severe mitochondrial ultrastructural injury was observed in the brain tissues of the Hcy-treated group. The significant morphological changes consisted primarily of a significant swelling of the mitochondrial matrix (Fig. 1C,F). In some instances, mitochondrial swelling was accompanied by a disruption of membrane integrity. These observations were corroborated by the mitochondrial ultrastructural analyses that documented elevated mitochondrial injury scores both in the brain cortex and the DG region of the hippocampus after the administration of Hcy (Fig. 1C,D).

### Hcy promotes cytochrome c (Cyt c) translocation and inhibits mitochondrial complex activities

**Figure 2:**
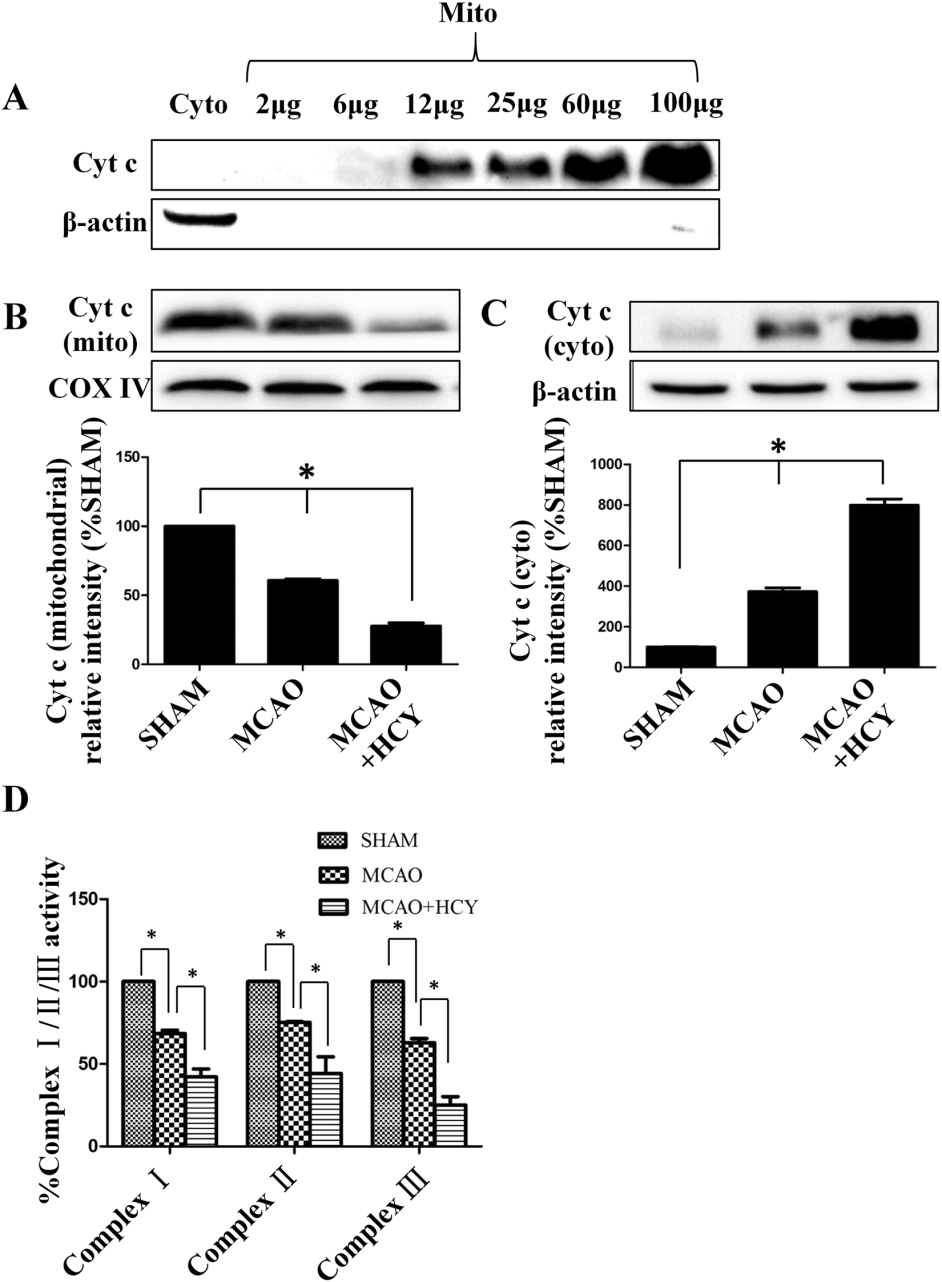
Effect of Hcy on Cyt c cytoplasmic translocation and ETC complex activities. (**A**) Purity of isolated mitochondria as evaluated by western blot analysis using Cyt c. (**B**,**C**) Western blots were performed to test Cyt c translocation. Cytochrome c oxidase IV (COX IV), a marker for mitochondria, was used as a loading control for the mitochondrial (mito) fraction. β-actin served as a loading control for the cytosolic (cyto) fraction. (**D**) Effects of Hcy on the activities of mitochondrial respiratory chain complexes (Complex I, II, and III). Measurements of mitochondrial respiratory activities were performed by using brain mitochondrial isolates from SHAM, MCAO, and Hcy-treated (MCAO + HCY) animals. Values are given as the means ± SD of five independent experiments. *P < 0.05. Full length blots of Fig. 2A–C are included in Supplementary Figs S1–3.

Cyt c is a pivotal protein that resides in mitochondria as a component of mitochondrial respiration and as an apoptosis initiator^26^. To further analyze the impact of Hcy on mitochondria-mediated apoptosis, western blotting was performed to test the Cyt c level in purified mitochondria (Fig. 2A). As shown in Fig. 2B and C, compared to that in the SHAM group, a significant reduction in the protein levels of Cyt c in the mitochondrial fraction and a concomitant increase in Cyt c levels in the cytosolic fraction were observed after MCAO injury. Furthermore, Hcy significantly increased the release of mitochondrial Cyt c into the cytosol, resulting in reduced mitochondrial Cyt c levels compared with that in the MCAO group (*P* < 0.05).﻿

To evaluate whether Hcy could affect mitochondrial function, the enzyme activities of electron transfer chain (ETC) Complexes I–III were assessed. As shown in Fig. 2D, there was a significant difference in the activities of Complex I–III among the three experimental groups. Specifically, all three enzyme activities in the Hcy group were significantly decreased than in the MCAO group (*P* < 0.05).

### Hcy increases 8-hydroxy-deoxyguanosine (8-OHdG) levels in the hippocampus and the brain cortex

**Figure 3:**
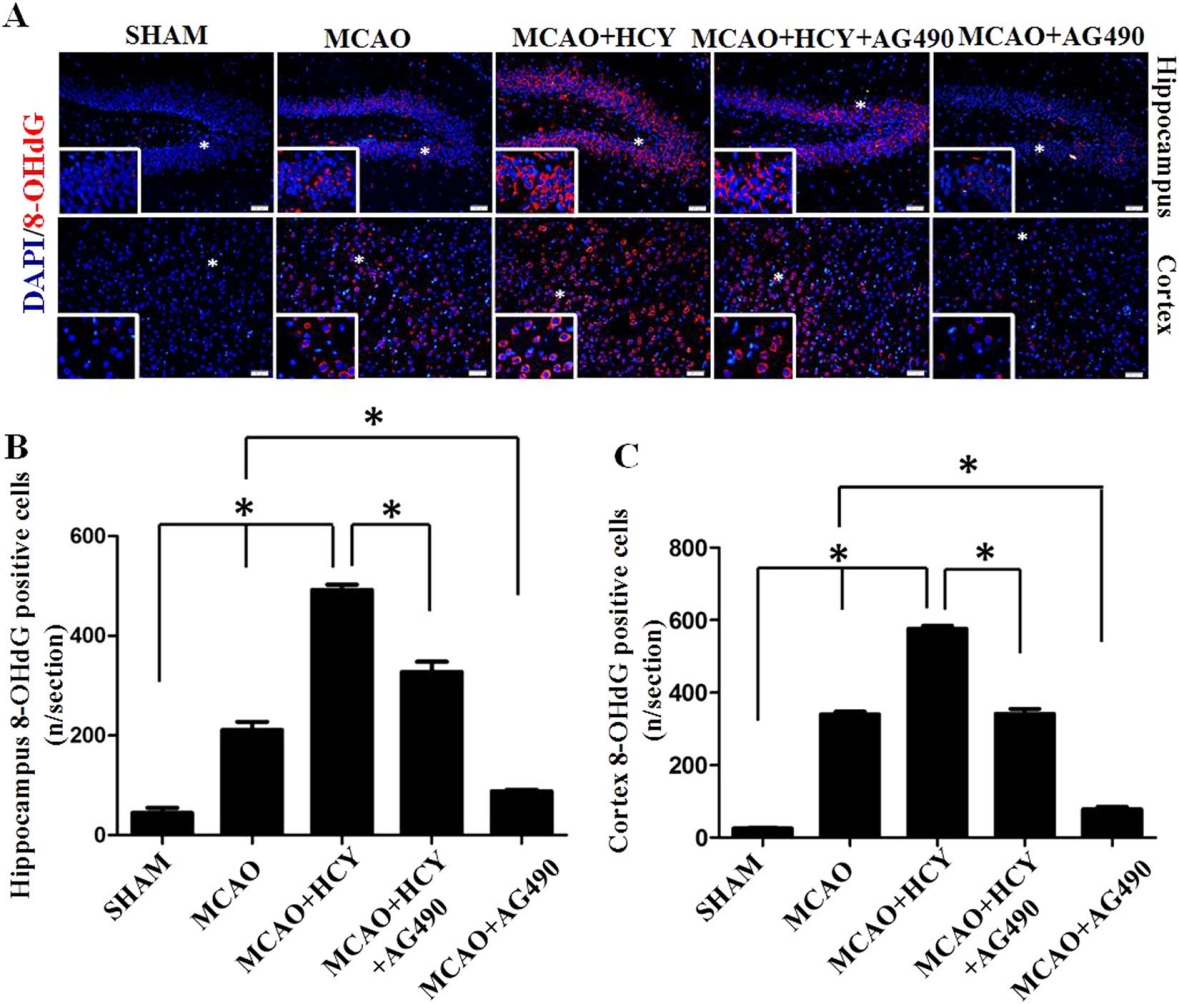
Hcy increases 8-OHdG levels in the hippocampus and the brain cortex after ischemia injury. (**A**) Representative images illustrating the changes in 8-OHdG levels in the brain cortex and hippocampus. Double staining for 8-OHdG (red) and DAPI (blue) was performed. (**B**,**C**) Quantitative assessment of 8-OHdG positive cells per field in the hippocampus and brain cortex. Six rats in each group and 10 fields for each rat were examined. Data are expressed as the means ± SD. *P < 0.05, Scale bars = 50 μm.

As alterations in ETC complexes can potentially increase electron leakage and cause oxidative stress, we next evaluated mitochondrial oxidative damage. 8-OHdG represents a sensitive marker of oxidative DNA damage and oxidative stress. In this study, we examined whether the injured neural cells were oxidatively stressed by high levels of Hcy, using 8-OHdG as a marker. Immunofluorescence staining of 8-OHdG showed that the numbers of 8-OHdG positive cells were significantly higher both in the brain cortex and the DG region of the hippocampus in the MCAO + HCY group compared with that in the MCAO group alone (Fig. 3).

### Hcy increases mitochondrial phosphorylated STAT3 (pSTAT3) expression in the ischemic brain

**Figure 4:**
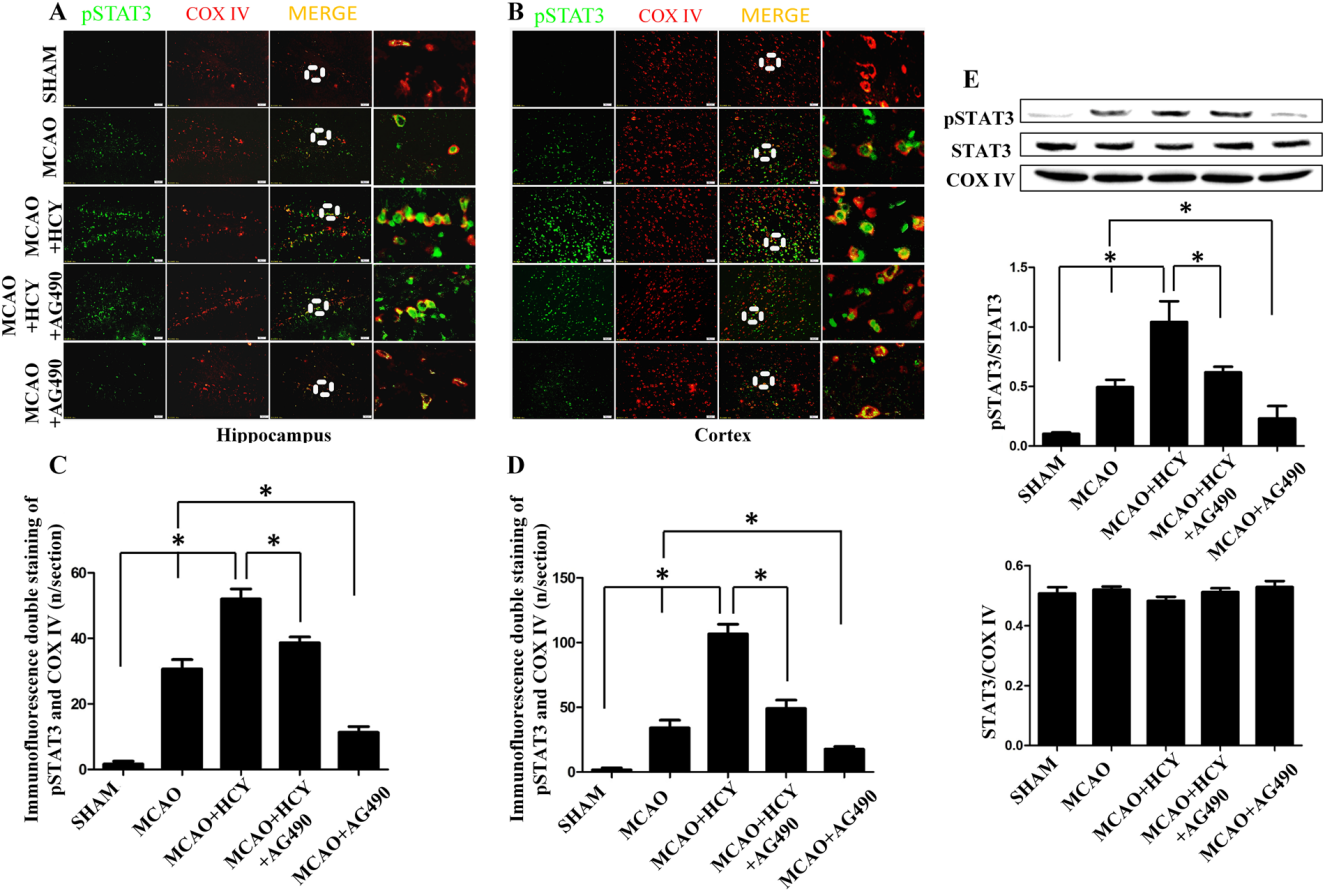
Hcy increases pSTAT3 expression in mitochondria. (**A**,**B**) Fluorescence immunohistochemistry for pSTAT3 and COX IV (a mitochondrial marker) 24 h following MCAO injury. Representative images of the mitochondrial marker COX IV (red) and STAT3 (green) staining in the DG region of the rat hippocampus and the cerebral ischemic cortex. Overlays demonstrate the colocalization of pSTAT3 and COX IV. Scale bars = 50 µm. (**C**,**D**) Quantitative assessment of pSTAT3/COX IV positive cells in the rat brain hippocampus and the cortex (n = 6/group, 10 fields/brain). (**E**) Representative western blot for pSTAT3. Bar graphs show semiquantitative levels of pSTAT3 as determined by band density analysis. n = 5 per group. *P < 0.05. The entire of membrane pictures of Fig. 4E were presented in the Supplementary Fig. S4.

To determine whether pSTAT3 localizes to mitochondria in the ischemic brain, we conducted double immunohistochemical staining for pSTAT3 and the mitochondrial marker cytochrome c oxidase subunit IV (COX IV) in paraffin embedded sections of ischemia. As detected by fluorescence microscopy, pSTAT3 and COX IV were colocalized both in the hippocampus (Fig. 4A) and the brain cortex (Fig. 4B).

A marked increase in the number of pSTAT3 and COX IV double-positive cells was observed in the MCAO group vs. the SHAM group, *P* < 0.05. Injections of Hcy significantly further increased the number of pSTAT3 and COX IV-positive cells in the hippocampus (Fig. 4C, *P* < 0.05) and the cerebral ischemic cortex (Fig. 4D, *P* < 0.05). However, following AG490 treatment, a specific inhibitor of activated Jak2 which is primarily responsible for STAT3 phosphorylation, this increase was partly reversed. In addition, no differences in total STAT3 concentrations were detected among all experimental groups.

To further confirm that Hcy activated mitochondrial STAT3 in the ischemic brain, we analyzed the expression of pSTAT3 at 24 h after brain injury by western blotting. Similar results were obtained from mitochondria isolated from the ischemic brain. Ischemia injury resulted in a significant increase in pSTAT3 protein expression compared with that in the SHAM group (*P* < 0.05). The protein level of pSTAT3 was further increased in the MCAO + HCY group compared with that in the MCAO group (*P* < 0.05). In addition, the pSTAT3 protein expression was significantly inhibited in MCAO + AG490 group compared with that in the MCAO group (Fig. 4E, *P* < 0.05).

### Crosstalk between ROS and pSTAT3 in Neuro2a (N2a) cells

**Figure 5:**
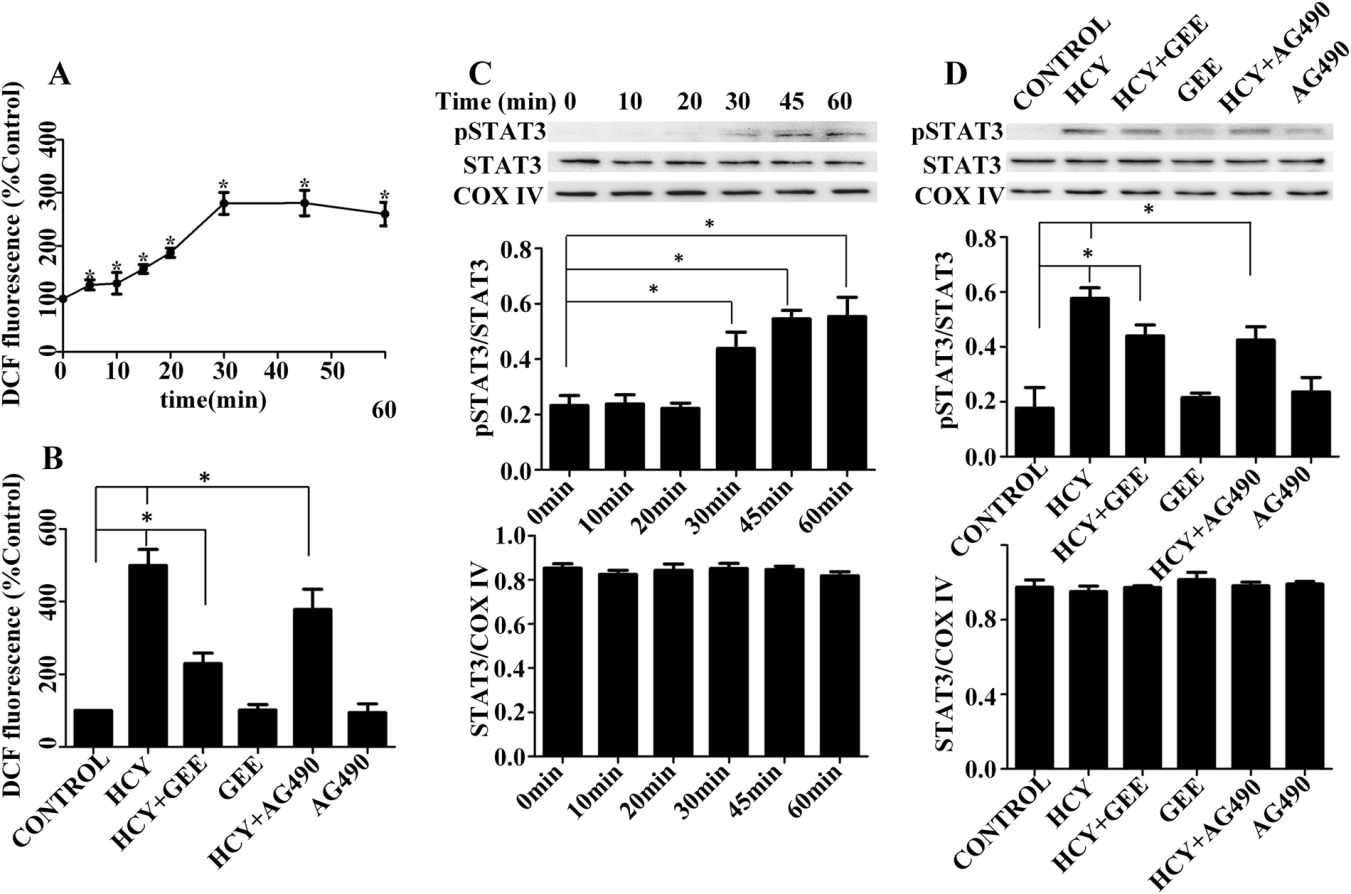
Crosstalk between ROS and pSTAT3 in Hcy-treated N2a cells. (**A**) Time course of Hcy-induced ROS generation. Cells were loaded with DCFH-DA (10 µM) for 30 min prior to incubation with 500 μM Hcy. Formation of ROS in cells was determined at the indicated time points (0 min, 5 min, 10 min, 15 min, 20 min, 30 min, 45 min, and 60 min) after exposure to 500 μM Hcy. *P < 0.05 compared with the basal value. (**B**) ROS levels in the presence of the JAK2 inhibitor (AG490) and a ROS inhibitor (GEE). The cells were respectively preincubated with 5 mM GEE for 30 min and 500 μM AG490 for 60 min prior to incubation with 500 μM Hcy. The ROS levels were evaluated by performing DCF measurement at 30 min after Hcy treatment. Data are presented as the relative increase compared with the control (100%) (n = 5). *P < 0.05. (**C**) Time course of Hcy-induced STAT3 phosphorylation. The cells were incubated for the indicated time points with 500 μM Hcy. The protein levels were detected by western blot. *P < 0.05 compared with the basal value. (**D**) pSTAT3 protein expression in Hcy-treated N2a cells following the quenching of endogenous ROS levels with GEE and AG490 treatment. Following incubation of the cells with 500 μM Hcy for 60 min, pSTAT3 was detected by western blot. The western blots were typical of three independent experiments. *P < 0.05. The entire of membrane pictures of Fig. 5C,D were presented in the Supplementary Figs S5 and S6.

To assess whether Hcy also induced ROS accumulation in N2a cells, we measured total cellular ROS levels using the fluorescent probe2′,7′-dichlorofluorescin diacetate (DCFH-DA). We found that the level of intracellular ROS was increased in N2a cells treated with 500 μM D,L-Hcy, as shown in Fig. 5A, as revealed by the increase in DCF fluorescence. Furthermore, Hcy induced the formation of ROS in a time-dependent manner. DCF fluorescence began to increase by 5 min and reached a maximal level at 30 min. These results suggested that the treatment of N2a cells with Hcy caused an immediate increase in ROS levels. In addition, pre-treatment with 5 mM glutathione reduced ethyl ester (GEE, a ROS scavenger) significantly suppressed Hcy-induced ROS formation (Fig. 5B). However, treatment with GEE alone did not markedly alter DCF fluorescence in the absence of Hcy.

We next tested the hypothesis that Hcy-induced pSTAT3 might similarly be localized to the mitochondria *in vitro*. Mitochondrial fractions were isolated from Hcy-treated and non-treated N2a cells and then total STAT3 as well as pSTAT3 were immunoblotted. Consistent with the *in vivo* data, mitochondrial STAT3 phosphorylation was also activated in Hcy-treated N2a cells, compared with that in non-treated control cells (*P* < 0.05). As shown in Fig. 5C, Hcy increased pSTAT3 levels in a time-dependent manner. A significant increase in pSTAT3 levels was observed as early as 30 min after the initiation of Hcy treatment with no change in the total amount of STAT3 protein; in addition, treatments longer than 45 min did not appear to result in higher pSTAT3 levels (Fig. 5C). AG490 significantly suppressed Hcy- induced STAT3 activation; however, AG490 treatment alone did not markedly alter the levels of pSTAT3 in the absence of Hcy (Fig. 5D).

To explore the potential crosstalk between ROS and pSTAT3, we measured (i) pSTAT3 by quenching endogenous ROS levels with GEE or (ii) ROS levels in the presence of a JAK2 inhibitor (AG490). To study the role of pSTAT3 in the formation of ROS induced by Hcy, the cells were treated with AG490. As shown in Fig. 5D, AG490 significantly inhibited ROS formation after Hcy treatment, which was consistent with the *in vivo* experiment. In the brain cortex and the DG region of the hippocampus after ischemic damage, AG490 treatment also significantly reduced the Hcy-mediated elevation in 8-OHdG levels (Fig. 3). We next conducted the converse studies by analyzing the levels of pSTAT3 in the presence of GEE. As shown in Fig. 5D, quenching ROS significantly reduced the elevated levels of pSTAT3 caused by Hcy in N2a cells. Taken together, our results demonstrate that the inhibition of pSTAT3 (via AG490) decreased ROS levels and quenching ROS (via GEE) inhibited pSTAT3, thereby suggesting a positive feedback loop between these factors. Further, the maximum response of ROS generation occurred 30 min after Hcy treatment, with the increase initiating after just 5 min following treatment. In contrast, pSTAT3 levels began to increase 30 min following Hcy treatment. Thus, the activation of STAT3 might constitute a downstream event of ROS generation.

## Discussion

Hcy represents a key metabolic intermediate in the metabolism of sulfur amino acids. Both *in vitro* and *in vivo* studies have shown that Hcy is toxic to neuronal cells. However, very little has been reported on the effects and neurotoxic mechanisms of Hcy on the neural cells in the ischemic brain. In the present study, we found that Hcy treatment caused a significant mitochondrial ultrastructural injury in the ischemic cortex and hippocampus, promoted Cyt c translocation, and inhibited ETC Complex I–III activities. Further study showed that Hcy increased mitochondrial pSTAT3 and ROS levels in both N2a cells and the ischemic brain and that crosstalk occurred between ROS and pSTAT3 in the Hcy-treated cells. These results indicated that oxidative stress, mitochondrial STAT3 overactivation, and their interplay may be involved in the mechanism of Hcy-induced mitochondrial injury in the hippocampus and cortex of ischemic brains.

Hcy-induced changes in oxidative stress have largely been studied in the neural cell cultures and the animal models of some neurodegenerative diseases such as Alzheimer and Parkinson^27–30^. For example, Hcy has been found to induce neuronal cell injury and neurological dysfunction via oxidative stress in an Alzheimer’s disease model. Hcy directly increased the neurotoxicity of amyloid ß by inducing ROS formation^30^. However, it is unclear whether oxidative injury also plays an important role in Hcy-treated ischemic brains. In the current study, both *in vitro* studies of N2a neural cells and research in animal models of focal cerebral ischemia demonstrated that a high Hcy level led to an increase in oxidative stress. What’s worth mentioning is that experimental studies do not consistently support the role of oxidative stress in the brain injury. Gomez *et al*. has concluded that mildly high Hcy decreases ROS generation in isolated brain mitochondria from normal adult rats under non-stressed conditions^31^. Possibly, this discrepancy results from the presence or absence of a mutual influence of co-morbid hyperhomocystinemia to other pathological damage (such as brain ischemia, Alzheimer’s or Parkinson’s disease). Further, our results also showed that the mitochondria suffer more severe damage, including changes in morphology and function, in ischemic brains following Hcy treatment. Conversely, mitochondria are considered the primary intracellular sources of ROS as well as the targets of oxidative stress^32, 33^. Thus, Hcy may cause mitochondrial dysfunction by raising ROS levels.

The decline in the activities of mitochodrial ETC complexes has been reported to involve the pathological mechanism of Hcy-associated diseases. For instance, Timkova *et al*. recently investigated the effect of HHcy on rat heart function, and found that Hcy-induced myocardial injury was accompanied by a significant reduction in the activities of ETC complexes II and III, whereas activity of the complex I was unchanged^34^. However, our findings revealed that the elevated Hcy levels caused mitochondrial dysfunction, which was displayed as a significant decrease in all three enzyme activities of ETC complexes I- III in ischemic brains. Interestingly, Folbergrová *et al*. demonstrated that Hcy could selectively inhibit the activity of complex I in the cerebral cortex of immature rats following seizures since the activity of complex II remained unaffected^15^. Therefore, it seems that the impact of Hcy on the activities of ETC complexes varies according to the animal model used and the context of the study.

STAT3 represents another factor that has been demonstrated to play a role in the process of cerebral ischemia and reperfusion injury^35, 36^. Notably, for over two decades it has been widely accepted that STAT3-dependent biology is due to its potency as a transcription factor capable of regulating the expression of many hundreds of genes associated with cell proliferation, differentiation, and survival^35, 37^. Thus, the finding that STAT3 also localizes to mitochondria has opened a new avenue to discover non-classical functions of this protein. The presence of STAT3 in mitochondria (mitoStat3), first discovered in heart, has been further confirmed in different cells and tissues in a number of follow up studies^21–25^. Recent findings have also supported new aspects of mitoStat3 in regulating mitochondrial metabolism and cellular function^38^. However, although it has been shown that the cellular pSTAT3 level was induced following transient cerebral ischemia in rat^36^, no evidence has been presented regarding whether cerebral ischemia stimulates the expression of pSTAT3 in mitochondria. In the current study, double staining with COX IV and pSTAT3 demonstrated that increased pSTAT3 expression could be detected in the mitochondria of the ischemic cortex and hippocampus in the MCAO or Hcy-treatment models. Conversely, the enhancement of mitoStat3 expression consequent to ischemic injury or Hcy treatment was significantly decreased by AG490 administration. To our knowledge, this is thus the first study to demonstrate that mitoStat3 activation is associated with the pathogenesis of stroke and Hcy neurotoxicity.

However, the role of STAT3 in ischemic pathogenesis is complicated and conflicting data have been reported across similar stroke models. Some studies have found that STAT3 activation is associated with increased cell survival whereas others have related it to cell death. Further, several groups have found that the JAK2-STAT3 pathway was activated by hypoxic preconditioning, rhEPO, or other novel compounds, which improved neurological recovery and/or decreased cell death after experimental stroke^39, 40^. Accordingly, blockage of JAK2-STAT3 phosphorylation via AG490 or WP1066 (an analog of AG490) induced cell death and worsened functional performance^39–41^. In contrast, some studies have shown that activation of the JAK2-STAT3 pathway instead leads to decreased cerebral recovery and that blocking this pathway through the administration of AG490 or a STAT3 siRNA led to improved neurological outcomes such as decreased infarction volume, neuronal damage, and apoptosis^42^. In the present study, we found that the levels of mitoStat3 and mitochondrial injury were significantly increased in both the brain cortex and hippocampus after MCAO and these effects were magnified by increased Hcy levels. In our models, the overactivation of mitoStat3 caused by Hcy appeared to be harmful for the ischemic brain. Thus, whether pSTAT3 activation is beneficial or destructive appears to depend on the cellular location of pSTAT3 expression or the extent of protein activation.

In recent years, several lines of evidence have indicated that the JAK2/STAT3 signal pathway is hyperactivated in cellular and animal models of oxidative stress injury, suggesting an important role of this signaling pathway in regulating oxidative stress responses^43–45^. It has been verified that H_2_O_2_ treatment significantly increased the level of pSTAT3 in a dose-dependent manner in peripheral blood human lymphocytes, fibroblasts, A-431 carcinoma cells, and in human umbilical vein endothelial cells^43, 44, 46^. Further evidence has shown that ROS prevented the dephosphorylation of STAT3, whereas induced STAT3 phosphorylation was completely abolished by treatment with the ROS inhibitor N-acetyl L-cysteine^47^. In addition, Lei *et al*. demonstrated that transient focal cerebral ischemia-induced STAT3 activation might be mediated by ROS production on the basis of evidence that STAT3 activation is blocked by a ROS scavenger in a dose-dependent manner^45^. However, the role of ROS in inducing mitochondrial STAT3 activation has not been elucidated. The role of mitoStat3 in the regulation of mitochondrial ROS is also unclear. It was recently reported that the JAK2-STAT3 pathway up-regulates oxidative stress and increases ROS levels and cell death^44, 46, 48^. Here, our results indicated that a crosstalk occurred between ROS and mitoStat3, which may constitute an important mechanism for the neuronal injury caused by Hcy. Furthermore, ROS levels began to increase at 5 min in Hcy-treated cells; in contrast, pSTAT3 levels began to increase after 30 min following Hcy treatment. Thus, it would appear that ROS generation represents one of the early changes caused by Hcy whereas activation of STAT3 might comprise a downstream event of ROS generation.

In conclusion, we demonstrate that elevated Hcy levels caused mitochondrial dysfunction, which was displayed as a significant decrease in respiratory chain activities, increased ROS production, and mitochondrial swelling in the hippocampus and cortex following ischemic injury. Over-expression of pSTAT3 induced by Hcy was localized to the mitochondria and was correlated to the regulation of ROS production. Early ROS generation may further induce mitochondria-localized STAT3 activation. Therefore, our results suggest that lowering Hcy levels or interference with the biochemical mechanisms involved in oxidative stress may represent potential therapeutic strategies in ischemic brain damage.

## Materials and Methods

### Materials

D,L-Homocysteine, 2′,7′-Dichlorofluorescin diacetate (DCFH-DA), Glutathione reduced ethyl ester (GEE), AG490, and Bovine Serum Albumin (BSA) were purchased from Sigma-Aldrich (St. Louis, MO, USA). The primary antibodies (mouse monoclonal antibody against STAT3, rabbit monoclonal antibody against phosphorylated STAT3 at Tyr705 (pSTAT3), and mouse monoclonal antibody against β-actin) and the secondary antibodies (fluorescein isothiocyanate (FITC)-conjugated goat anti-rabbit IgG, tetraethyl rhodamine isothiocyanate (TRITC)-conjugated goat anti-mouse IgG, horseradish peroxidase-linked anti-rabbit and anti-mouse IgG) were purchased from Cell Signaling Technology (Danvers, MA, USA). Mouse monoclonal antibodies against 8-hydroxy-deoxyguanosine (8-OHdG), Cytochrome c oxidase subunit IV (COX IV), and Cytochrome c (Cyt c) were purchased from Abcam (Cambridge, MA, USA). The assay kits for electron transport chain Complex I, Complex II and Complex III were purchased from Nanjing Jiancheng Bioengineering Institute (Nanjing, China). Ethylene diamine tetraacetic acid (EDTA), Trypsin-EDTA solution and Lysis buffer were purchased from Beyotime Biotech (Wuhan, China). 4′,6-diamidino-2-phenylinedole, dihydrochloride (DAPI) was purchased from Solarbio (Beijing, China). Dulbecco’s modified Eagle’s medium (DMEM) and fetal bovine serum (FBS) was purchased from Gibco-BRL (Paisley, UK). NC membranes, and immobilon western chemiluminescent horseradish peroxidase substrate were from Millipore (Bedford, MA, USA).

### N2a Cell Culture and treatment

The N2a cell line was obtained from Huaxi Xu (Biomedical Research, Xiamen University, Xiamen, China) and cultured as previously described^49^. Briefly, the cells were grown in DMEM with 10% FBS and 1% penicillin/streptomycin and incubated at 37 °C with 5% CO_2_/95% air in a humidified atmosphere. Media was renewed every 2 days. The N2a cells were respectively pretreated with the ROS inhibitor GEE (5 mM) for 30 min and AG490 (500 μM) for 60 min and then with Hcy (500 μM). Cell cultures were exposed to D,L-Hcy (500 μM) for different lengths of time immediately prior to the end of the experiment.

### Measurement of ROS levels in N2a cells

The formation of ROS in N2a cells was measured using the probe DCFH-DA. The cells were seed at a density of 1 × 10^6^/well with 2 ml DMEM culture medium into 6-well plates, pre-incubated with 10 μM DCFH-DA for 30 min in the dark, and then treated with Hcy, GEE (5 mM), or AG490 (500 μM). DCF intensity was measured immediately by inversion fluorescence microscopy (excitation 470–490 nm/emission 510–550 nm; Olympus XI81; Tokyo, Japan).

### Animals and experimental design

We purchased 100 adult male Sprague-Dawley (SD) rats (180 g–220 g) (Grade SPF, Certificate Number SCXK (jing) 2012–0001) from Vital River Laboratory Co. Ltd. (Beijing, China). Use of animals and experimental procedures were conducted in accordance with the Guide for the Care and Use of Laboratory Animals published by the National Institutes of Health (NIH publication No. 80-23, revised 1996). The Tianjin Medical University Animal Ethics Committee approved the experimental protocols in this study (Study Number: TMUaMEC 2012016). The rats were randomly allocated into five groups: sham operation control group (SHAM), MCAO group, MCAO plus Hcy group (MCAO + HCY), MCAO, Hcy plus AG490 group (MCAO + HCY + AG490) and MCAO plus AG490 group (MCAO + AG490). The vehicle and D,L-Hcy (1.6 mg/kg/day) were administered by tail vein injection for 21 days prior to SHAM or MCAO operation and up to 24 h after surgery. The AG490 (3 mg/kg) was administered by intraperitoneal injection prior to 10 min of SHAM or MCAO operation.

### Surgical procedures for MCAO

Transient focal ischemia-reperfusion was induced by MCAO in the subject animals and the modified Longa method was used to assess the neurological deficit^50^. Briefly, rats were anesthetized by intraperitoneal injection of 1% sodium pentobarbital (40 mg/kg). A midline incision was made at the skin of neck area and the external, internal, and common carotid arteries were carefully exposed. The left external carotid artery was tied off and the internal carotid artery was closed. A nylon intraluminal suture (length, 18–20 mm, diameter, 0.24 mm) was advanced through the left internal carotid artery to the origin of the middle cerebral artery. One hour after the operation, the suture was slowly and carefully withdrawn to allow reperfusion. The sham-operated controls underwent similar surgical procedures with the exception of suture insertion. The animals were separately euthanized at 24 h after reperfusion for the subsequent experiments.

### Transmission electron microscopy and evaluation of mitochondrial injury

Ultrastructural (electron microscopy) evaluation was performed as previously described^51^. Briefly, the cortex and hippocampus of the left brain hemispheres were sliced into 1-mm^3^ portions on ice and immersed in an isotonic fixative consisting of 4% paraformaldehyde, 2.5% glutaraldehyde, and 0.1 M sucrose in 0.1 M phosphate buffer (pH = 7.4). The issue pieces were then post fixed in the same medium containing 1% osmium tetroxide and subsequently dehydrated in a graded ethanol series (50%, 70%, 80%, 90%, and 100%). The dehydrated samples were embedded in epoxy resin and embedded fragments were then sliced and stained with uranyl acetate and lead citrate and examined under a HT-7700 transmission electron microscope (Hitachi, Tokyo, Japan).

Mitochondrial injury was blindly assessed using a scoring system that is based on the typical ultrastructural features of mitochondria attendant to the progressive stages of cellular injury, as previously described^52, 53^. Briefly, five grids of each group and four fields of per grid were randomly selected by the reviewers. The standards for the evaluation of mitochondrial injury from grade 0 to 4 were as follows: grade 0, normal mitochondria (mitochondria appeared highly dense with well-organized cristae); grade 1, early swelling as manifested by early clearing of matrix density and separation of cristae (a large amorphous matrix density and a linear density are present); grade 2, more marked swelling as manifested by further clearing of matrix density and separation of cristae; grade 3, more extensive mitochondrial swelling with disruption of cristae; grade 4, severe mitochondrial swelling with disruption of cristae and rupture of inner and outer mitochondrial membranes.

### Mitochondrial isolation and purity assessment

Rats were decapitated and the brain was rapidly removed and placed in ice-cold phosphate buffer; alternately, the N2a cells were harvested and washed three times with ice-cold phosphate buffer. Then, the excised brain tissues or N2a cells were homogenized in ice cold isolation buffer (300 mM sucrose, 1 mM EDTA, 1.5 mM MgCl_2_, and 20 mM HEPES, pH 7.4). The tissue samples were minced with scissors and then the minced brain or cells were transferred into a glass homogenizer that was then fill with ice cold isolation buffer with subsequent homogenization 10–30 times. The homogenized tissue or cells was then centrifuged at 1,000 × g for 10 min at 4 °C and the resulting supernatants were centrifuged at 15,000 × g for 10 min at 4 °C. The pellets were washed with ice-cold mitochondria lysis buffer and spun at 20,000 × g for 10 min at 4 °C. The final pellets were suspended in lysis buffer containing 1% Triton X-100 and were mitochondrial-rich lysate fractions. The supernatant was further centrifuged at 20,000 × g for 10 min at 4 °C to obtain cytoplasmic proteins. The concentrations of the respective proteins were measured according to the Bradford method using BSA as a standard.

The purity of the mitochondrial fraction was assessed by western blotting, demonstrating that the fraction was absent of a cytosolic marker (β-actin) and enriched in a mitochondrial marker (Cyt c).

### Measurement of the activities of mitochondrial Complex I-III

Mitochondrial activity of Complex I (NADH dehydrogenase) was measured according to a previous method^54^. Briefly, the decrease in NADH absorbance at 340 nm that occurs when ubiquinone (CoQ1) is reduced to form ubiquinol was measured. The reaction medium was composed of 20 mM phosphate buffer, pH 7.2, 2 mM KCN, 5 mM MgCl_2_, 60 μM decylubiquinone, and 0.15 mM NADH in a final volume of 1 ml. The reaction was initiated with the addition of 100 μg mitochondrial protein. The activity of Complex I was presented as the increase compared with the control (100%).

Mitochondria activity of Complex II (succinate-ubiquinone oxidoreductase) was determined as the succinate-driven malonate-sensitive rate of 2,6-dichlorophenolindophenol (DCPIP) reduction at 600 nm^48^. The assay reaction mixture contained 40 mM potassium phosphate pH 7.2, 0.1 mM EDTA, 2 mM KCN, 20 mM succinate, 60 μM DCPIP dye, 10 μM rotenone, and 50 μM CoQ2 in final volume of 1 ml. The reaction was initiated with the addition of 100 μg mitochondrial protein. The activity of Complex II was presented as the increase compared with the control (100%).

Mitochondria activity of Complex III (ubiquinone-cytochrome-c oxidoreductase) was measured by monitoring the reduction of oxidized Cyt c (III) to reduced Cyt c at 550 nm as previously described^55^. The mixture solution containing 35 mM PBS, pH 7.2, 5 mM MgCl_2_, 2 mM KCN, 2.5 mg/ml BSA, 60 μM reduced decylubiquinol, and 50 μM Cyt c in a final volume of 1 ml. The reaction was initiated with the addition of 100 μg mitochondrial protein. The activity of Complex III was presented as the increase compared with the control (100%).

### Immunofluorescence staining

Immunofluorescence staining was performed as described previously^56^. Briefly, the rats were anesthetized and perfused with 200 ml 0.9% saline and 4% paraformaldehyde, the brains were removed, fixed in 4% paraformaldehyde and then embedded in paraffin. Sections were de-waxed in xylene for 15 min, hydrated in a graded alcohol series (100%, 90%, 75% and 50%) and treated with 3% H_2_O_2_ for 10 min at room temperature. Subsequently the tissues underwent antigen retrieval by incubating the sections for 6 min in a solution of citric acid at 90 °C. The brain sections were blocked with goat serum for 45 min at 37 °C, incubated with the primary antibodies (pSTAT3 1:100, Cox IV 1:500, 8-OHdG 1:500) overnight at 4 °C and incubated with the FITC-conjugated goat anti-rabbit or TRITC-conjugated goat anti-mouse secondary antibodies (1:100) for 1 h at 25 °C, DAPI (10 μg/ml) was used to dye the nuclei before 10 min of mounting. The fluorescence microscope (IX81; Olympus, Tokyo, Japan) was used to observe and photograph. Positive cells were counted by Image Pro Plus 6.0.

### Western blotting

Rats were euthanized at 24 h after reperfusion. Western blotting was performed to analyze protein expression in the mitochondria or cytosol from ischemic brains and N2a cells. In brief, the brain tissues or cells were homogenized in RIPA buffer (20 mM TRIS-HCl pH 7.5, 150 mM NaCl, 1 mM EDTA, 1% Triton-X100, 0.5% sodium deoxycholate, 1 mM PMSF and 10 µg/ml leupeptin), incubated on ice for 30 min, and centrifuged at 14,000 g for 10 min at 4 °C. The supernatants were collected, and protein concentration was detected with BSA as a standard according to the Bradford method^57^. Equal amounts of protein from each sample were separated by 12% sodium dodecyl sulfate-polyacrylamide gel electrophoresis and transferred to nitrocellulose blotting membranes (NC membranes; Millipore) by wet electrical transfer method. Subsequently, the membranes were blocked with 5% milk (Sigma) in 1× Tris buffered saline Tween for 1 h at room temperature, followed by incubation with the primary antibodies (Cyt c 1:5,000; pSTAT3 1:1,000; STAT3 1:1,000; β-actin 1:10,000; COX IV 1:1,000) overnight at 4 °C and incubated with the goat anti-rabbit or goat anti-mouse secondary antibodies (1:2,000) for 1 h at 25 °C. Then the blots were developed by immobilon western chemiluminescent horseradish peroxidase substrate and observed using a ChemiDocTM XRS + Imaging System (Bio-Rad, Hercules, CA, USA). The protein levels were quantified by densitometric analysis using NIH ImageJ 1.61 Software (National Institutes of Health, Bethesda, MD, USA).

### Statistical analysis

Statistical analysis was performed using SPSS, version 19.0 (SPSS, Chicago, IL, USA). The results are presented as the means ± standard deviation ($\overline{x}\pm s$). Differences between means were evaluated by one-way analysis of variance (ANOVA) followed by Least Significant Difference (LSD) multiple range test. *P* < 0.05 was considered to be statistically significant.

## Electronic supplementary material

supplementary information

## References

[1] Homocysteine Studies Collaboration. Homocysteine and risk of ischemic heart disease and stroke: a meta-analysis. *JAMA* **288**, 2015–2022 (2002). 10.1001/jama.288.16.2015 12387654

[2] Clarke R (1998). Folate, vitamin B_12_, and serum total homocysteine levels in confirmed Alzheimer disease. Arch Neurol.

[3] Allain P (1995). Sulfate and cysteine levels in the plasma of patients with Parkinson’s disease. Neurotoxicology.

[4] Zoccolella S (2008). Elevated plasma homocysteine levels in patients with amyotrophic lateral sclerosis. Neurology.

[5] Valentino F (2010). Elevated cerebrospinal fluid and plasma homocysteine levels in ALS. Eur J Neurol.

[6] Zhao Y (2016). Homocysteine aggravates cortical neural cell injury through neuronal autophagy overactivation following rat cerebral ischemia-reperfusion. Int J Mol Sci.

[7] Okamoto K, Kondo-Okamoto N (2012). Mitochondria and autophagy: critical interplay between the two homeostats. Biochim Biophys Acta.

[8] Baek SH (2014). Modulation of mitochondrial function and autophagy mediates carnosine neuroprotection against ischemic brain damage. Stroke.

[9] Li J (2012). Reperfusion promotes mitochondrial dysfunction following focal cerebral ischemia in rats. PloS One.

[10] Racay P (2009). Mitochondrial calcium transport and mitochondrial dysfunction after global brain ischemia in rat hippocampus. Neurochem Res.

[11] Solenski NJ, diPierro CG, Trimmer PA, Kwan AL, Helm GA (2002). Ultrastructural changes of neuronal mitochondria after transient and permanent cerebral ischemia. Stroke.

[12] Wei YH, Lu CY, Lee HC, Pang CY, Ma YS (1998). Oxidative damage and mutation to mitochondrial DNA and age-dependent decline of mitochondrial respiratory function. Ann N Y Acad Sci.

[13] Shigenaga MK, Hagen TM, Ames BN (1994). Oxidative damage and mitochondrial decay in aging. Proc Natl Acad Sci USA.

[14] Niizuma K (2010). Mitochondrial and apoptotic neuronal death signaling pathways in cerebral ischemia. Biochim Biophys Acta.

[15] Folbergrova J (2007). Mitochondrial complex I inhibition in cerebral cortex of immature rats following homocysteic acid-induced seizures. Exp Neurol.

[16] Wen TC, Peng H, Hata R, Desaki J, Sakanaka M (2001). Induction of phosphorylated-Stat3 following focal cerebral ischemia in mice. Neurosci Lett.

[17] Suzuki S (2001). Phosphorylation of signal transducer and activator of transcription-3 (Stat3) after focal cerebral ischemia in rats. Exp Neurol.

[18] Justicia C, Gabriel C, Planas AM (2000). Activation of the JAK/STAT pathway following transient focal cerebral ischemia: signaling through Jak1 and Stat3 in astrocytes. Glia.

[19] Wegrzyn J (2009). Function of mitochondrial Stat3 in cellular respiration. Science.

[20] Garama DJ, White CL, Balic JJ, Gough DJ (2016). Mitochondrial STAT3: powering up a potent factor. Cytokine.

[21] Gough DJ (2009). Mitochondrial STAT3 supports Ras-dependent oncogenic transformation. Science.

[22] Yang, R. *et al*. Mitochondrial Ca(2)(+) and membrane potential, an alternative pathway for Interleukin 6 to regulate CD4 cell effector function. *eLife* **4**, doi:10.7554/eLife.06376 (2015). 10.7554/eLife.06376 PMC4447996 25974216

[23] Lachance C, Goupil S, Leclerc P (2013). Stattic V, a STAT3 inhibitor, affects human spermatozoa through regulation of mitochondrial activity. J Cell Physiol.

[24] Shulga N, Pastorino JG (2012). GRIM-19-mediated translocation of STAT3 to mitochondria is necessary for TNF-induced necroptosis. J Cell Sci.

[25] Heusch G, Musiolik J, Gedik N, Skyschally A (2011). Mitochondrial STAT3 activation and cardioprotection by ischemic postconditioning in pigs with regional myocardial ischemia/reperfusion. Circ Res.

[26] Yang G (2009). Mitochondrial dysfunction resulting from loss of cytochrome c impairs radiation-induced bystander effect. Br J Cancer.

[27] Currò M (2014). Toxic effects of mildly elevated homocysteine concentrations in neuronal-like cells. Neurochem Res.

[28] Bhattacharjee N, Borah A (2016). Oxidative stress and mitochondrial dysfunction are the underlying events of dopaminergic neurodegeneration in homocysteine rat model of Parkinson’s disease. Neurochem Int.

[29] Bukharaeva E, Shakirzyanova A, Khuzakhmetova V, Sitdikova G, Giniatullin R (2015). Homocysteine aggravates ROS-induced depression of transmitter release from motor nerve terminals: potential mechanism of peripheral impairment in motor neuron diseases associated with hyperhomocysteinemia. Front Cell Neurosci.

[30] Ho PI (2001). Homocysteine potentiates beta-amyloid neurotoxicity: role of oxidative stress. J Neurochem.

[31] Gomez J (2011). Methionine and homocysteine modulate the rate of ROS generation of isolated mitochondria *in vitro*. J Bioenerg Biomembr.

[32] Kowaltowski AJ, de Souza-Pinto NC, Castilho RF, Vercesi AE (2009). Mitochondria and reactive oxygen species. Free Radic Biol Med.

[33] Cui H, Kong Y, Zhang H (2012). Oxidative stress, mitochondrial dysfunction, and aging. J Signal Transduct.

[34] Timkova V (2016). Effects of mild hyperhomocysteinemia on electron transport chain complexes, oxidative stress, and protein expression in rat cardiac mitochondria. Mol Cell Biochem.

[35] Raible DJ, Frey LC, Brooks-Kayal AR (2014). Effects of JAK2-STAT3 signaling after cerebral insults. JAKSTAT.

[36] Choi JS (2003). Upregulation of gp130 and STAT3 activation in the rat hippocampus following transient forebrain ischemia. Glia.

[37] Zhao J, Li G, Zhang Y, Su X, Hang C (2011). The potential role of JAK2/STAT3 pathway on the anti-apoptotic effect of recombinant human erythropoietin (rhEPO) after experimental traumatic brain injury of rats. Cytokine.

[38] Yang R, Rincon M (2016). Mitochondrial Stat3, the need for design thinking. Int J Biol Sci.

[39] Zhu H, Zou L, Tian J, Du G, Gao Y (2013). SMND-309, a novel derivative of salvianolic acid B, protects rat brains ischemia and reperfusion injury by targeting the JAK2/STAT3 pathway. Eur J Pharmacol.

[40] Zhou TF, Yu JG (2013). Recombinant human erythropoietin attenuates neuronal apoptosis and cognitive defects via JAK2/STAT3 signaling in experimental endotoxemia. J Surg Res.

[41] Sun JJ, Liu Y, Ye ZR (2008). Effects of P2Y1 receptor on glial fibrillary acidic protein and glial cell line-derived neurotrophic factor production of astrocytes under ischemic condition and the related signaling pathways. Neurosci Bull.

[42] Satriotomo I, Bowen KK, Vemuganti R (2006). JAK2 and STAT3 activation contributes to neuronal damage following transient focal cerebral ischemia. J Neurochem.

[43] Simon AR, Rai U, Fanburg BL, Cochran BH (1998). Activation of the JAK-STAT pathway by reactive oxygen species. Am J Physiol.

[44] Duan W (2013). New role of JAK2/STAT3 signaling in endothelial cell oxidative stress injury and protective effect of melatonin. PloS One.

[45] Lei C (2011). Reactive oxygen species scavenger inhibits STAT3 activation after transient focal cerebral ischemia-reperfusion injury in rats. Anesth Analg.

[46] Carballo M (1999). Oxidative stress triggers STAT3 tyrosine phosphorylation and nuclear translocation in human lymphocytes. J Biol Chem.

[47] Machida K (2006). Hepatitis C virus triggers mitochondrial permeability transition with production of reactive oxygen species, leading to DNA damage and STAT3 activation. J Virol.

[48] Tawfik A (2005). Hyperglycemia and reactive oxygen species mediate apoptosis in aortic endothelial cells through Janus kinase 2. Vascul Pharmacol.

[49] Li W (2015). Folic Acid Inhibits Amyloid beta-peptide production through modulating DNA methyltransferase activity in N2a-APP cells. Int J Mol Sci.

[50] Longa EZ, Weinstein PR, Carlson S, Cummins R (1989). Reversible middle cerebral artery occlusion without craniectomy in rats. Stroke.

[51] Flameng W, Borgers M, Daenen W, Stalpaert G (1980). Ultrastructural and cytochemical correlates of myocardial protection by cardiac hypothermia in man. J Thorac Cardiovasc Surg.

[52] Crouser ED, Julian MW, Blaho DV, Pfeiffer DR (2002). Endotoxin-induced mitochondrial damage correlates with impaired respiratory activity. Crit Care Med.

[53] Kloner RA, Fishbein MC, Braunwald E, Maroko PR (1978). Effect of propranolol on mitochondrial morphology during acute myocardial ischemia. Am J Cardiol.

[54] Birch-Machin MA, Briggs HL, Saborido AA, Bindoff LA, Turnbull DM (1994). An evaluation of the measurement of the activities of complexes I-IV in the respiratory chain of human skeletal muscle mitochondria. Biochem Med Metab Biol.

[55] Torres-Mendoza CE, Albert A (1999). & de la Cruz Arriaga, M. J. Molecular study of the rat liver NADH: cytochrome c oxidoreductase complex during development and ageing. Mol Cell Biochem.

[56] Lee JY (2016). Microvesicles from brain-extract-treated mesenchymal stem cells improve neurological functions in a rat model of ischemic stroke. Sci Rep.

[57] Bradford MM (1976). A rapid and sensitive method for the quantitation of microgram quantities of protein utilizing the principle of protein-dye binding. Anal Biochem.

