# Supplementary material for: Homocysteine induces mitochondrial dysfunction involving the crosstalk between oxidative stress and mitochondrial pSTAT3 in rat ischemic brain
Source: Sci Rep. 2017 Jul 31;7:6932. doi: 10.1038/s41598-017-07112-z (PMC5537278; doi:10.1038/s41598-017-07112-z)
Supplement: Supplementary file 1 — supplementary information [file 41598_2017_7112_MOESM1_ESM.pdf]

Homocysteine induces mitochondrial dysfunction involving the crosstalk between oxidative stress and mitochondrial pSTAT3 in rat ischemic brain

Shuang Chen, Zhiping Dong, Yaqian Zhao, Na Sai, Xuan Wang, Huan Liu, Guowei Huang and Xumei Zhang*

Department of Nutrition and Food Science, School of Public Health, Tianjin Medical University, Tianjin, China

*Correspondence: Department of Nutrition and Food Science, School of Public Health, Tianjin Medical University, Tianjin 300070, China. E-mail: zhangxumei@tmu.edu.cn

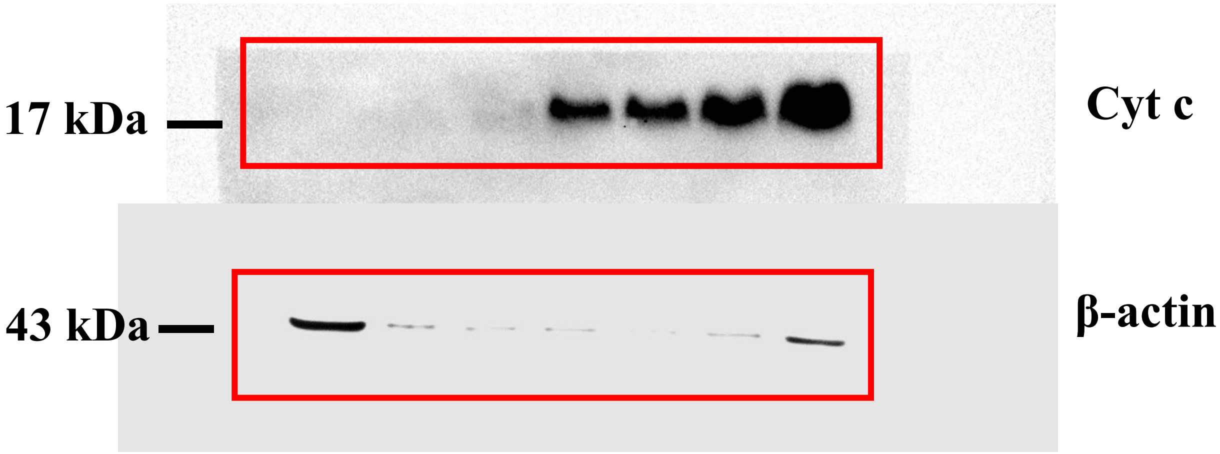

Supplementary Fig. S1 Full-length blots for blots shown in Fig.2A

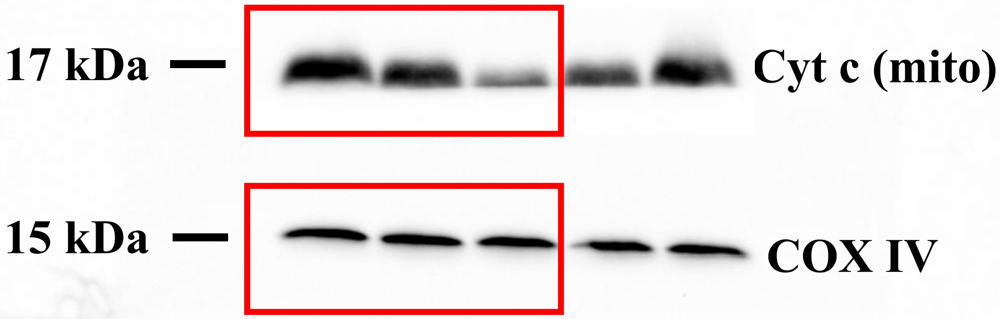

Supplementary Fig. S2 Full-length blots for blots shown in Fig.2B

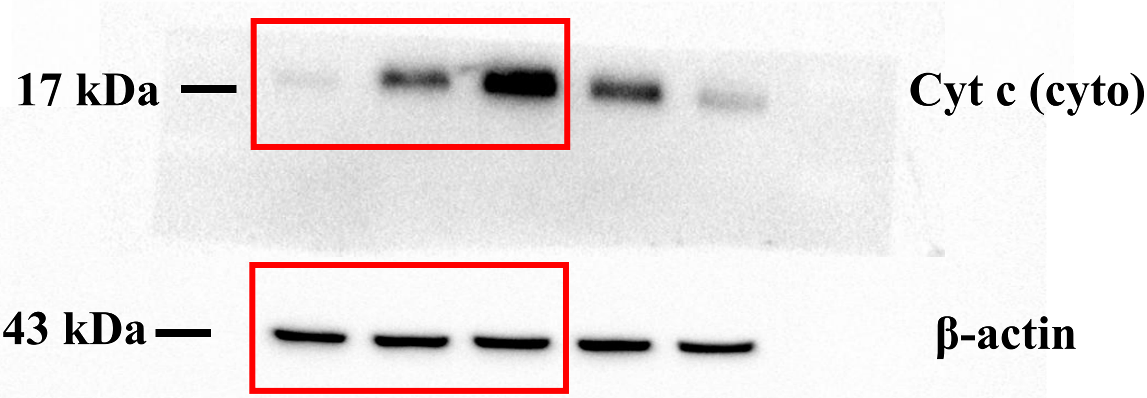

Supplementary Fig. S3 Full-length blots for blots shown in Fig.2C

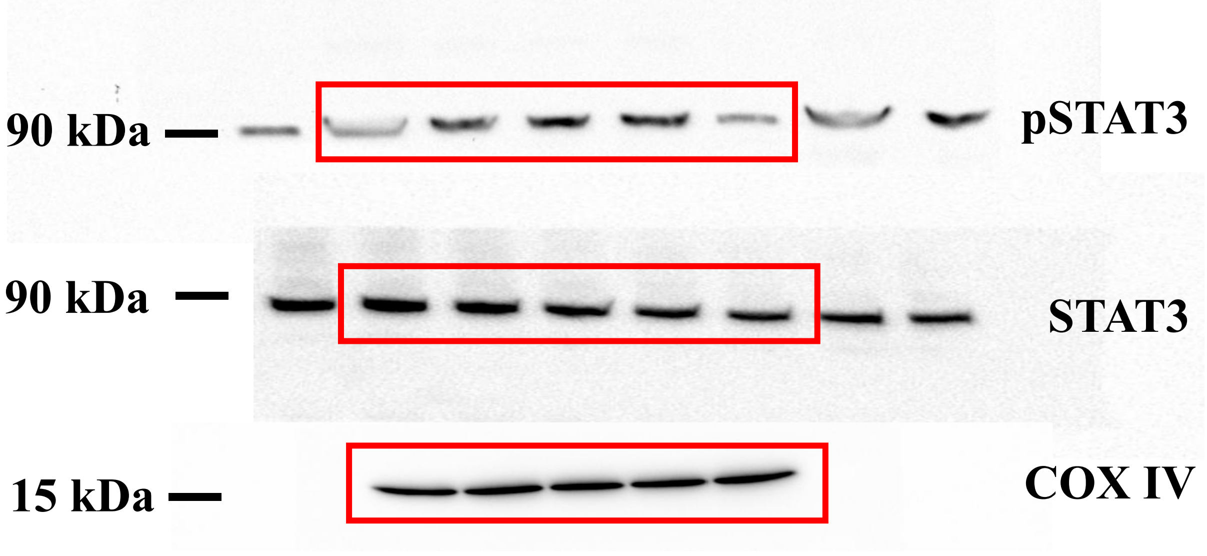

Supplementary Fig. S4 Full-length blots for blots shown in Fig.4E

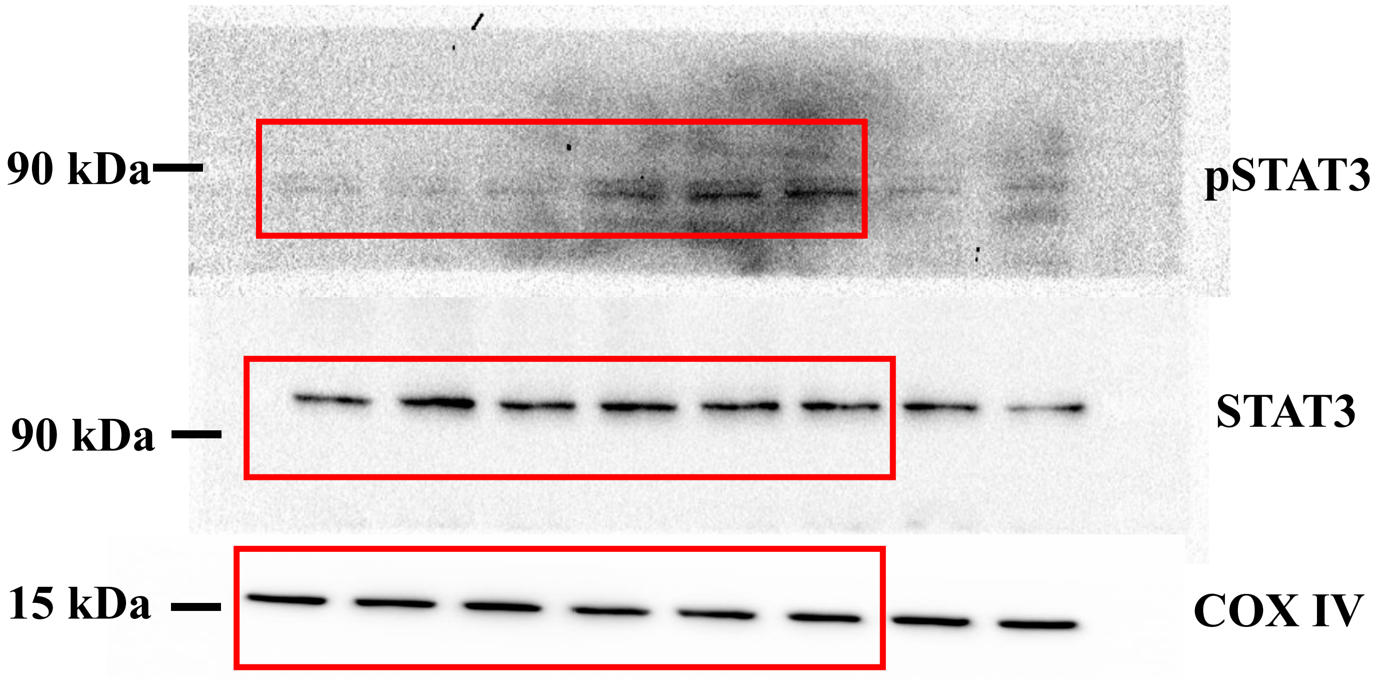

Supplementary Fig. S5 Full-length blots for blots shown in Fig.5C

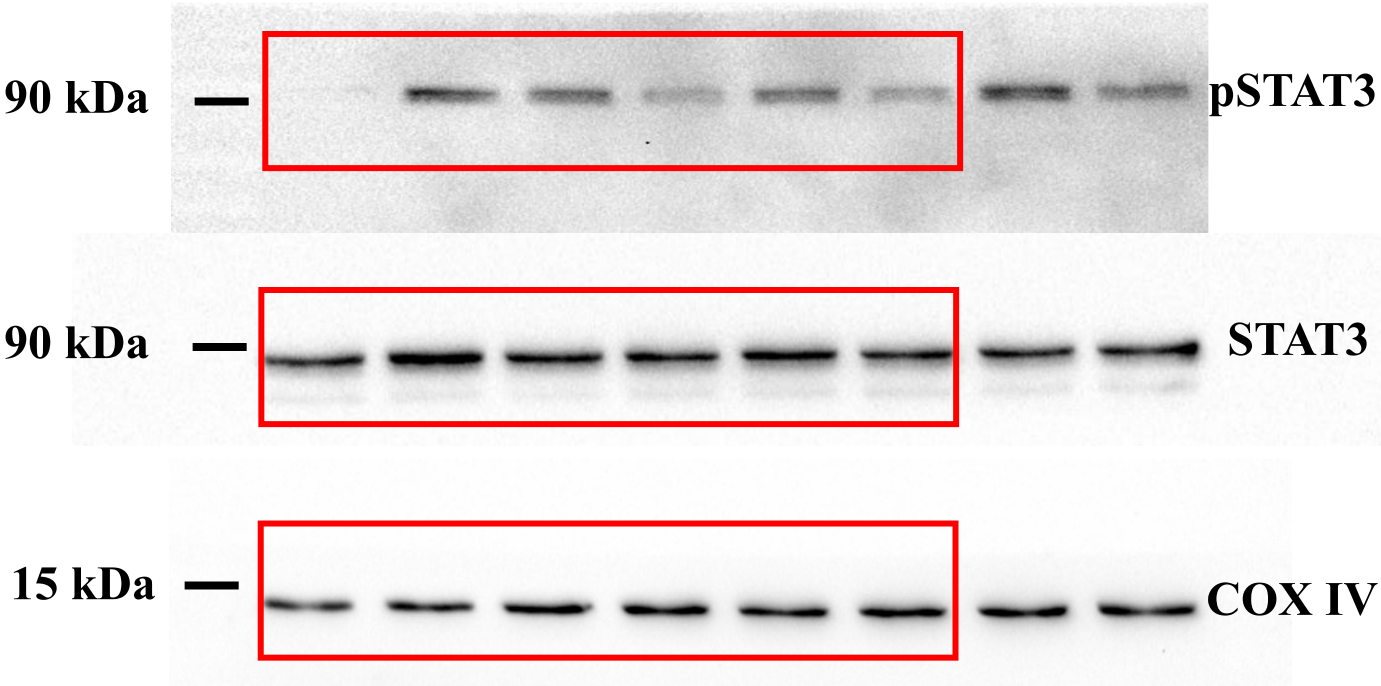

Supplementary Fig. S6 Full -length blots for blots shown in Fig.5D
